# Supplementary material for: Plasma metabolomics reveals the shared and distinct metabolic disturbances associated with cardiovascular events in coronary artery disease
Source: Nat Commun. 2024 Jul 8;15:5729. doi: 10.1038/s41467-024-50125-2 (PMC11231153; doi:10.1038/s41467-024-50125-2)
Supplement: Supplementary file 1 — Supplementary Information [file 41467_2024_50125_MOESM1_ESM.pdf]

Supplementary Materials

Supplementary Methods

Supplementary Table 1. Medications during follow-up period.

Supplementary Table 2. The Pearson partial correlation coefficients of metabolic pairs in network analyses.

Supplementary Table 3. The calibration performance of the prediction models.

Supplementary Table 4. Predictive value of plasma metabolites in patients with acute coronary syndrome.

Supplementary Table 5. Patient inclusion and exclusion criteria.

Supplementary Table 6. Power of differential metabolites discovery.

Supplementary Figure 1. The key metabolites combination included in the composite of cardiovascular events prediction model.

Supplementary Figure 2. The partial least squares discrimination analysis 3D score plots.

Supplementary Figure 3. The volcano plot of differential metabolite analyses.

Supplementary Figure 4. The key metabolites combination included in cardiovascular death prediction model.

Supplementary Figure 5. The key metabolites combination included in heart failure prediction model.

Supplementary Figure 6. The key metabolites combination included in myocardial infarction/stroke prediction model.

Supplementary Figure 7. The calibration curves of prediction models for the composite of cardiovascular events.

Supplementary Figure 8. The calibration curves of prediction models for cardiovascular death.

Supplementary Figure 9. The calibration curves of prediction models for heart failure.

Supplementary Figure 10. The calibration curves of prediction models for myocardial infarction/stroke.

Supplementary Figure 11. The distribution of the key metabolites combination for the composite of cardiovascular events across different disease subtypes.

Supplementary Figure 12. The distribution of the key metabolites combination for cardiovascular death across different disease subtypes.

Supplementary Figure 13. The distribution of the key metabolites combination for heart failure across different disease subtypes.

Supplementary Figure 14. The distribution of the key metabolites combination for myocardial infarction /stroke across different disease subtypes.

Supplementary Figure 15. The flow chart of metabolomics data processing.

Supplementary Figure 16. Mirror plots of MS/MS match for acylcarnitines.

Supplementary Figure 17. Annotation of characteristic fragment ions in mass spectra of acylcarnitines.

Supplementary Figure 18. Mirror plots for MS/MS spectral match for phthalide and erucic acid.

Supplementary Methods

Patients in the discovery and validation set

Patients from site 1 (Qilu hospital of Shandong University, Jinan, China) were first randomly assigned to the discovery set (n = 334, 167 with cardiovascular events and 167 without any cardiovascular event), and the remained patients were assigned to the validation set (n = 240, 123 with cardiovascular events and 117 without any cardiovascular event). Then, patients from site 2 (Zibo Central hospital, Zibo, China) were assigned to the validation set (n = 92, 43 with cardiovascular events and 49 without any cardiovascular event). The baseline examination, including demographic characteristics, cardiovascular risk factors and medical history, previous medications, presenting symptoms and signs, electrocardiogram recordings, and admitting diagnosis were obtained from medical records. Anthropometric variables and blood pressure were assessed by qualified medical personnel in the participating sites.

Supplementary Table 1. Medications during follow-up period.

Medications	Discovery set					Validation set				
	Control (n=167)	Case (n=167)	CV death (n=82)	Heart failure (n=48)	MI/stroke (n=72)	Control (n=166)	Case (n=166)	CV death (n=76)	Heart failure (n=42)	MI/stroke (n=93)
**30 days after discharge**										
β receptor blockers, n (%)	127 (76.5)	125 (81.2)	54 (78.3)	39 (86.7)	58 (80.6)	119 (73.0)	101 (73.2)	33 (66.0)	18 (60.0)	67 (79.8)
ACEI/ARB, n (%)	87 (52.4)	89 (57.8)	38 (55.1)	26 (57.8)	42 (58.3)	89 (54.6)	74 (53.6)	33 (66.0)	18 (60.0)	39 (46.4)
Statins, n (%)	158 (95.2)	138 (89.6)	58 (84.1)	38 (84.4)	66 (91.7)	150 (92.0)	130 (94.2)	46 (92.0)	28 (93.3)	81 (96.4)
Aspirin, n (%)	155 (93.4)	140 (90.9)	61 (88.4)	41 (91.1)	65 (90.3)	145 (89.0)	131 (94.9)	45 (90.0)	29 (96.7)	83 (98.8)
**6 months after discharge**										
β receptor blockers, n (%)	117 (70.5)	104 (79.4)	34 (73.9)	33 (82.5)	51 (78.5)	116 (71.6)	92 (73.0)	25 (64.1)	15 (57.7)	61 (77.2)
ACEI/ARB, n (%)	72 (43.4)	57 (43.5)	20 (43.5)	18 (45.0)	26 (40.0)	82 (50.6)	62 (49.2)	23 (59.0)	17 (65.4)	36 (45.6)
Statins, n (%)	146 (88.0)	113 (86.3)	36 (78.3)	33 (82.5)	57 (87.7)	144 (88.9)	117 (92.9)	36 (92.3)	25 (96.2)	74 (93.7)
Aspirin, n (%)	147 (88.6)	113 (86.3)	36 (78.3)	33 (82.5)	56 (86.2)	141 (87.0)	120 (95.2)	36 (92.3)	25 (96.2)	77 (97.5)
**12 months after discharge**										
β receptor blockers, n (%)	113 (68.5)	92 (78.0)	24 (72.7)	31 (86.1)	48 (77.4)	113 (70.2)	88 (77.9)	21 (77.8)	14 (60.9)	60 (78.9)
ACEI/ARB, n (%)	67 (40.6)	47 (39.8)	12 (36.4)	18 (50.0)	24 (38.7)	75 (46.6)	52 (46.0)	18 (66.7)	11 (47.8)	33 (43.4)
Statins, n (%)	139 (84.2)	101 (85.6)	26 (78.8)	31 (86.1)	56 (90.3)	139 (86.3)	99 (87.6)	22 (81.5)	18 (78.3)	68 (89.5)
Aspirin, n (%)	143 (86.7)	99 (83.9)	26 (78.8)	30 (83.3)	51 (82.3)	135 (83.9)	105 (92.9)	25 (92.6)	21 (91.3)	72 (94.7)

Data are presented with n (%), Case indicates the composite of cardiovascular events.

ACEI = angiotensin converting enzyme inhibitors; ARB = angiotensin receptor blocker; CV = cardiovascular; MI = myocardial infarction.

Supplementary Table 2. The Pearson partial correlation coefficients of metabolic pairs in network analyses.

Network	X	Y	r	p
Cardiovascular death	Tetradecadienoylcarnitine (Car(14:2))	N,N-Dimethylarginine (ADMA)	0.156	0.183
Cardiovascular death	N-[3-(2-oxopyrrolidin-1-yl)propyl]acetamide	N,N-Dimethylarginine (ADMA)	0.595	1.791E-08
Cardiovascular death	Oxooctadecadienoylcarnitine (Car(18:3-O))	Tetradecadienoylcarnitine (Car(14:2))	0.481	1.233E-05
Cardiovascular death	Tetradecadienoylcarnitine (Car(14:2))	Hexadecanedioic acid	0.363	0.001
Cardiovascular death	3-Hydroxyoctanoic acid	Hexadecanedioic acid	0.337	0.003
Heart failure	N4-Acetylcytidine	Cystine	0.391	0.017
Heart failure	PC(22:2)	LPC(20:4)	0.589	1.274E-04
Heart failure	LPA(20:5)	PC(10:0/10:0)	0.488	0.002
Heart failure	LPC(O-16:0)	PC(8:0/8:0)	0.502	0.002
Heart failure	PC(10:0/10:0)	PC(8:0/8:0)	0.478	0.003
Heart failure	Cortisol	11beta-hydroxyandrost-4-ene-3,17-dione	0.524	0.001
Myocardial infarction/stroke	Suberic acid	3-(2,3,4-Trimethoxyphenyl)propanoic acid	0.397	0.001
Myocardial infarction/stroke	Palmitoleylcarnitine (Car(16:1))	Hexadecatrienoylcarnitine (Car(16:3))	0.262	0.037
Myocardial infarction/stroke	Oxooctanoylcarnitine (Car(8:1-O))	N4-Acetylcytidine	0.449	1.956 E-04
Myocardial infarction/stroke	Vanillylmandelic acid	N4-Acetylcytidine	0.577	5.905E-07
Myocardial infarction/stroke	3-(2,3,4-Trimethoxyphenyl)propanoic acid	Oxooctanoylcarnitine (Car(8:1-O))	0.249	0.048
Myocardial infarction/stroke	Hexadecatrienoylcarnitine (Car(16:3))	Oxooctanoylcarnitine (Car(8:1-O))	0.405	0.001

LPA = lysophosphatidic acid; LPC = lysophosphatidylcholine; PC = phosphatidylcholin

No multiple comparison was adjusted for myocardial infarction/stroke related metabolic pathways. The tests used for partial correlation coefficients were two-sided.

Supplementary Table 3. The calibration performance of the prediction models.

	The composite of cardiovascular events		Cardiovascular death		Heart failure		Myocardial infarction/Stroke	
**Model**	**χ^2**	**p**	**χ^2**	***P*-value**	**χ^2**	**p**	**χ^2**	**p**
The key metabolites combination	12.235	0.141	8.239	0.411	5.038	0.754	5.993	0.648
TIMI variables	7.417	0.492	9.861	0.275	3.679	0.885	6.693	0.570
TIMI variables & Metabolites	7.993	0.434	13.697	0.090	5.987	0.649	3.018	0.933
hs-cTnT	25.098	0.001	23.166	0.003	33.120	<0.001	20.823	0.008
hs-cTnT & Metabolites	16.132	0.041	6.540	0.587	5.141	0.742	4.526	0.807
NT-proBNP	4.470	0.812	13.078	0.109	3.341	0.852	7.866	0.447
NT-proBNP & Metabolites	8.421	0.393	4.215	0.837	1.664	0.990	0.902	0.999

The calibration performance of prediction models was evaluated by the Hosmer and Lemeshow goodness of fit (GOF) test (one-sided).

Supplementary Table 4. Predictive value of plasma metabolites in patients with acute coronary syndrome.

	The composite of cardiovascular events		Cardiovascular death		Heart failure		Myocardial infarction/Stroke	
**Model**	**AUC**	**p**	**AUC**	**p**	**AUC**	**p**	**AUC**	**p**
The key metabolites combination	0.65 (0.58, 0.70)*	-	0.70 (0.63, 0.77) †	-	0.88 (0.82, 0.93) ‡	-	0.63 (0.56, 0.70) §	-
TIMI variables ‖	0.63 (0.57, 0.69)	0.019	0.68 (0.60, 0.75)	0.019	0.68 (0.58, 0.77)	<0.001	0.62 (0.54, 0.70)	0.070
TIMI variables & Metabolites	0.70 (0.64, 0.75)		0.75 (0.69, 0.82)		0.90 (0.85, 0.94)		0.68 (0.61, 0.75)	
hs-cTnT	0.67 (0.61, 0.74)	0.546	0.72 (0.65, 0.79)	0.953	0.80 (0.73, 0.87)	0.035	0.65 (0.57, 0.72)	0.733
hs-cTnT & Metabolites	0.65 (0.59, 0.72)		0.72 (0.65, 0.79)		0.88 (0.83, 0.93)		0.63 (0.56, 0.71)	
NT-proBNP	0.71 (0.65, 0.76)	0.606	0.79 (0.73, 0.85)	0.518	0.88 (0.82, 0.92)	0.004	0.63 (0.55, 0.70)	0.201
NT-proBNP & Metabolites	0.72 (0.66, 0.77)		0.80 (0.74, 0.86)		0.94 (0.90, 0.97)		0.66 (0.59, 0.73)	

The above results only including patients with acute coronary syndrome at admission. The DeLong's test for two correlated ROC curves were two-sided test. Considering that only three tests were performed for each outcome, no multiple comparison correction was made for each test.

* The key metabolites combination of the composite of cardiovascular events were identified by Lasso algorithm, including cystine, hexenoylcarnitine (Car(6:1)), Oxooctadecadienoylcarnitine (Car(18:3-O)), Hexadecatrienoylcarnitine (Car(16:3)), 5-Acetylamino-6-amino-3-methyluracil (AAMU), erucic acid, suberic acid, Vanillylmandelic acid, Mandelic acid, 3-(2,3,4-Trimethoxyphenyl)propanoic acid, N4-Acetylcytidine, N-[3-(2-oxopyrrolidin-1-yl)propyl]acetamide, 3-Hydroxyoctanoic acid, Phthalide;

† The key metabolites combination of cardiovascular death were identified by Lasso algorithm, including Tetradecadienoylcarnitine (Car(14:2)), Car(18:3-O), Hexadecanedioic acid, Vanillylmandelic acid, N-[3-(2-oxopyrrolidin-1-yl)propyl]acetamide, 3-Hydroxyoctanoic acid, Phthalide, N,N-Dimethylarginine (ADMA), Homoarginine;

‡ The key metabolites combination of heart failure were identified by Lasso algorithm, including Cystine, N-Acetyl-arginine, Homoarginine, Erucic acid, N4-Acetylcytidine, 11beta-hydroxyandrost-4-ene-3,17-dione, Cortisol, LPA(20:5), LPC(20:4), LPC(O-16:0), PC(10:0/10:0), PC(22:2), PC(8:0/8:0);

§ The key metabolites combination of myocardial infarction/stroke were identified by Lasso algorithm, including 1,7-Dimethylxanthine, 3-(2,3,4-Trimethoxyphenyl) propanoic acid, Erucic acid, Car(16:3), Mandelic acid, N4-Acetylcytidine, Oxooctanoylcarnitine (Car(8:1-O)), Palmitoleylcarnitine (Car(16:1)), Suberic acid, Theobromine, Theophylline, Vanillylmandelic acid;

|| TIMI variables included age, current smoking, hypertension, diabetes mellitus, previous stroke, previous HF, previous PAD, previous PCI/CABG, and eGFR.

Supplementary Table 5. Patient inclusion and exclusion criteria.

**Inclusion criteria (all must be present)**
1. Hospitalized patients with the diagnosis of any type of coronary artery disease (stable angina, unstable angina, non-ST-segment elevation myocardial infarction or ST-segment elevation myocardial infarction).
2. Age ≥18 years of age.
3. Patient or guardian provided informed written consent.
4. Patient retained biological samples
**Exclusion criteria (all must be absent)**
1. Previous surgery, trauma or clinically evident bleeding (i.e. gastrointestinal, genitourinary) within the prior 2 weeks.
2. BIPass population with missing follow-up records in Qilu Hospital emergency follow-up cohort.

Supplementary Table 6. Power of differential metabolites discovery.

Events	Number of controls	Number of cases	Power
The composite of cardiovascular events	167	167	0.994
Cardiovascular death	167	82	0.950
Heart failure	167	48	0.844
Myocardial infarction/stroke	167	72	0.932

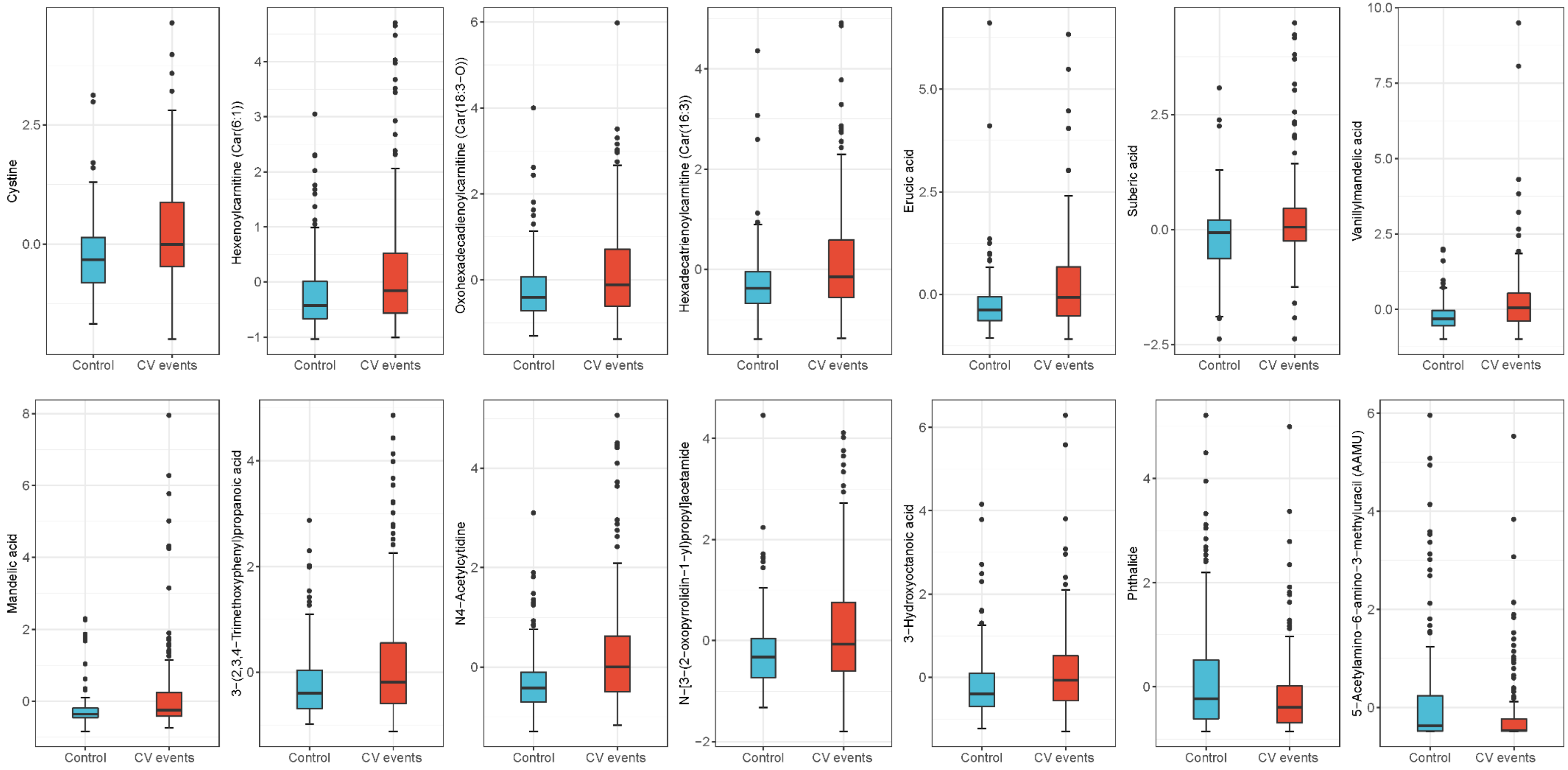

Supplementary Figure 1. The key metabolites combination included in the composite of cardiovascular events prediction model. CV = Cardiovascular.

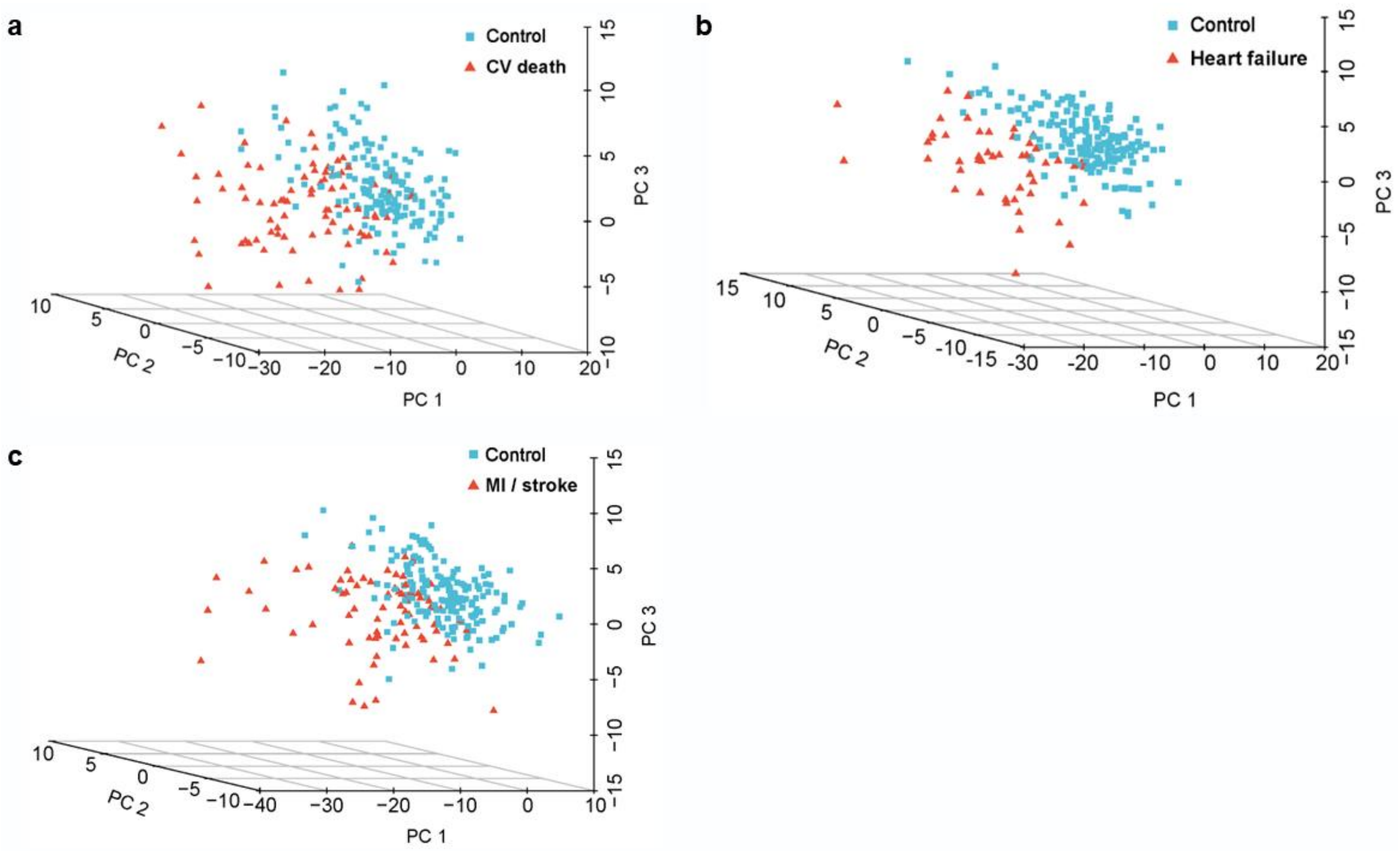

Supplementary Figure 2. The partial least squares discrimination analysis 3D score plots.

(a) Cardiovascular death; (b) Heart failure; (c) Myocardial infarction/stroke.

CV = Cardiovascular; MI = Myocardial infarction.

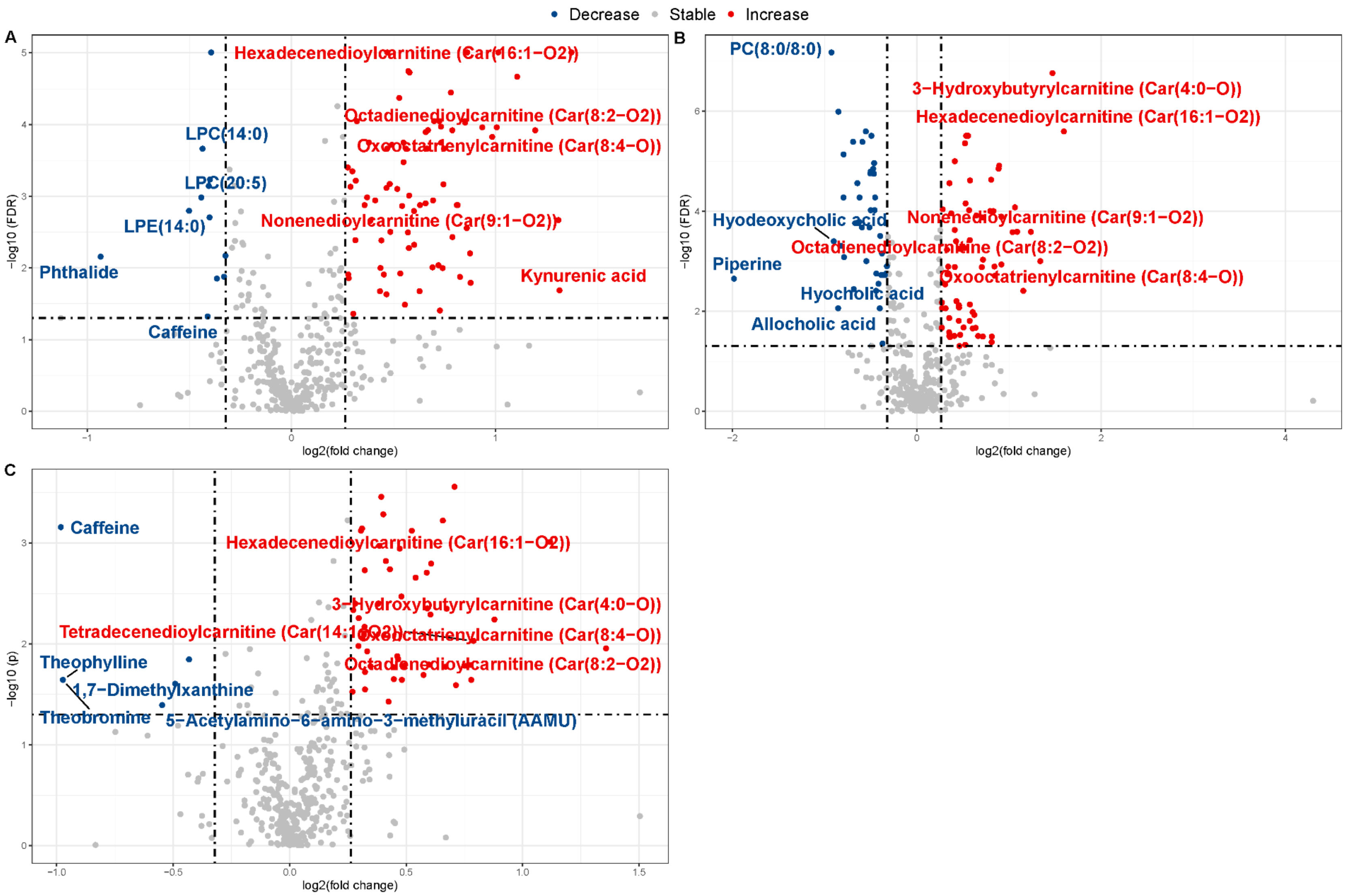

Supplementary Figure 3. The volcano plot of differential metabolite analyses.

(a) Cardiovascular death; (b) Heart failure; (c) Myocardial infarction/stroke.

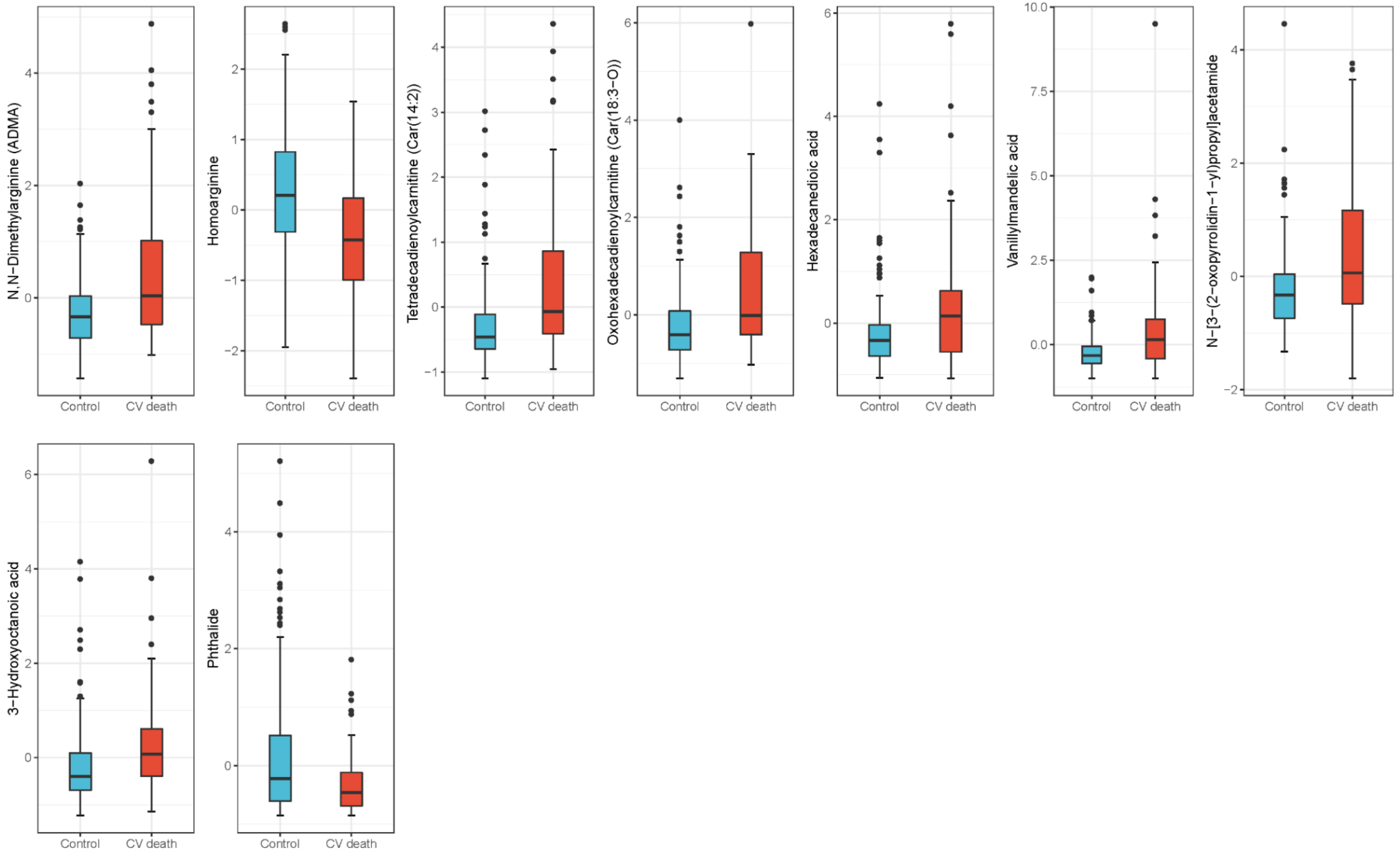

Supplementary Figure 4. The key metabolites combination included in cardiovascular death prediction model.

CV = Cardiovascular.

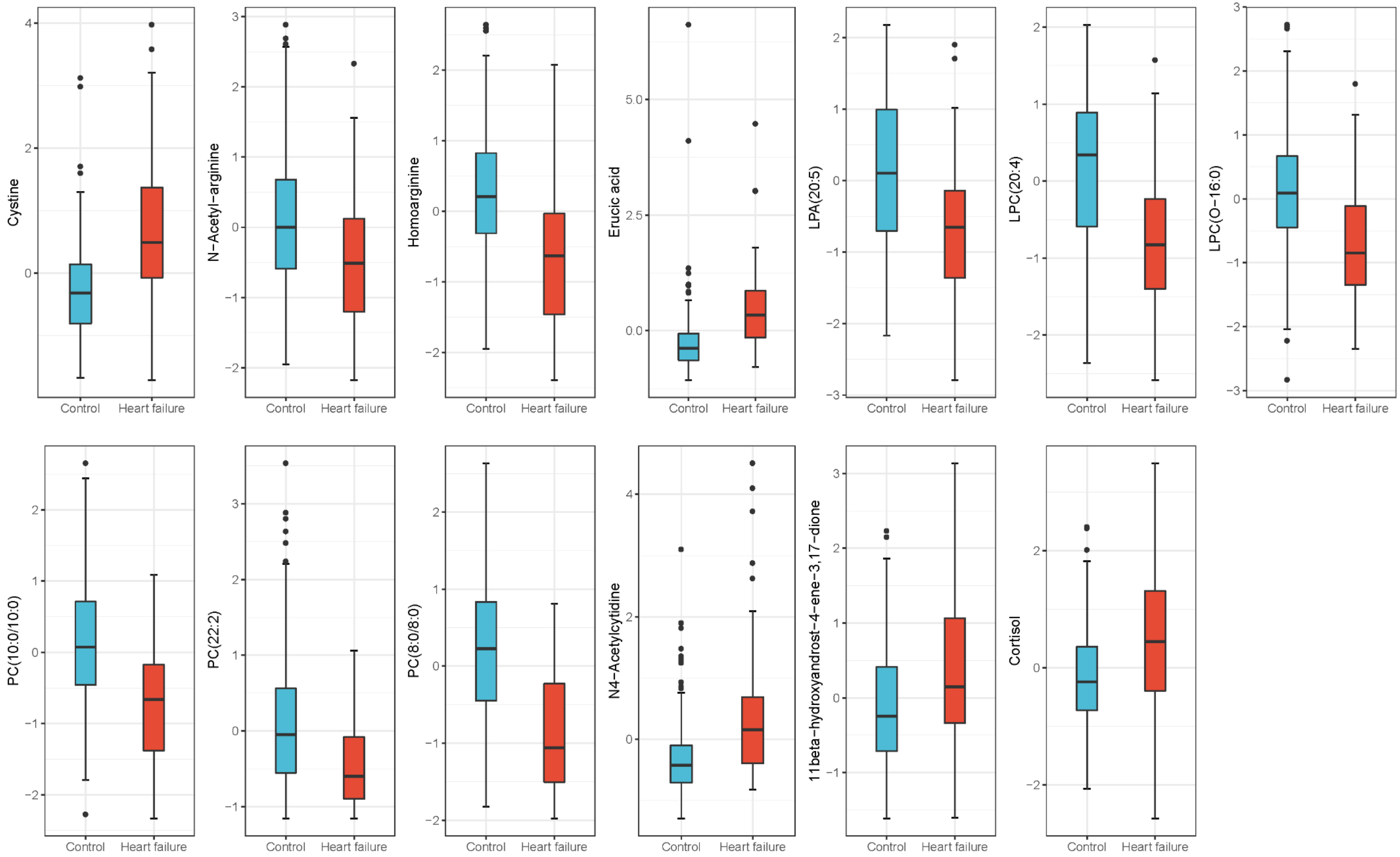

Supplementary Figure 5. The key metabolites combination included in heart failure prediction model.

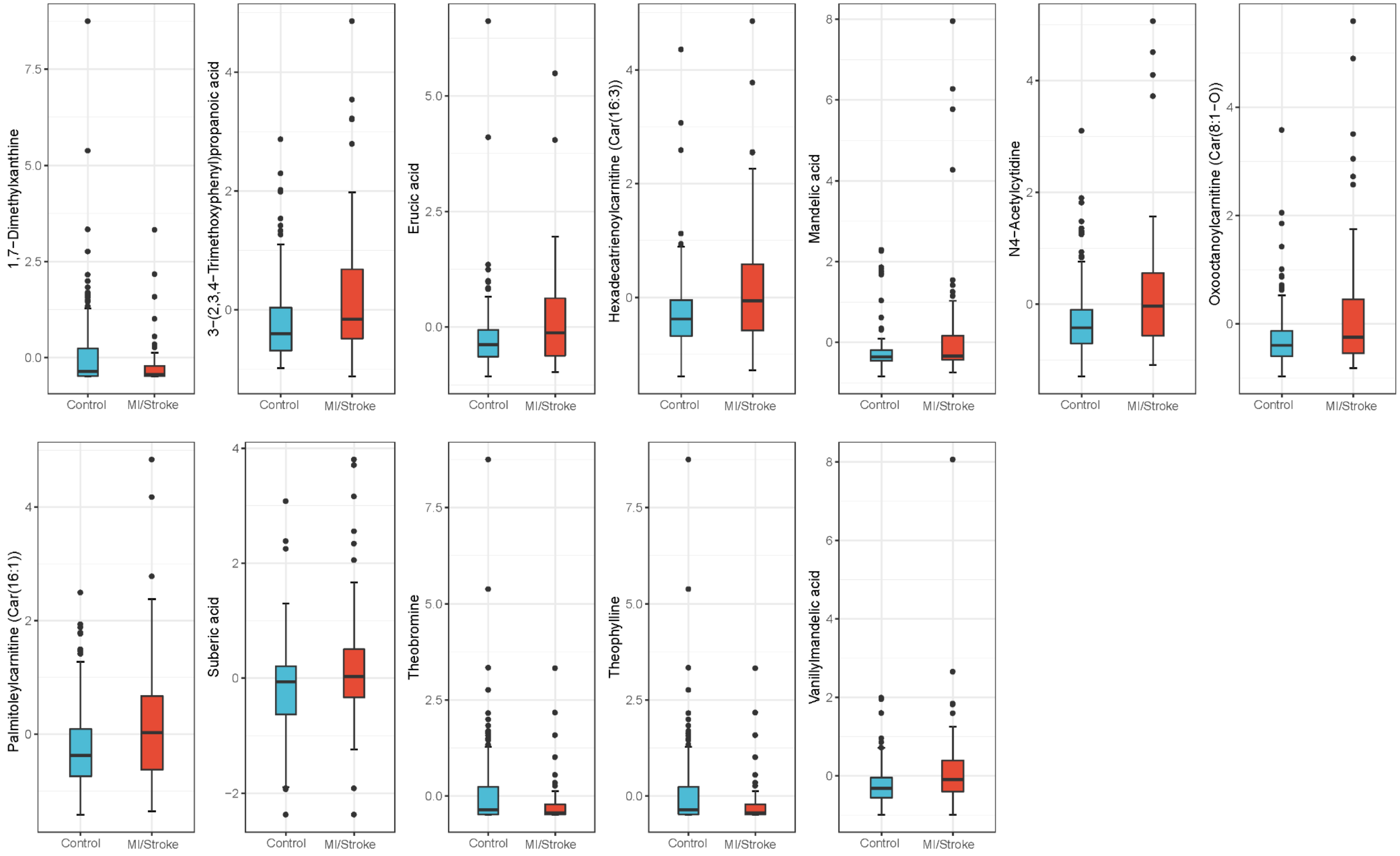

Supplementary Figure 6. The key metabolites combination included in myocardial infarction/stroke prediction model.

MI = Myocardial infarction.

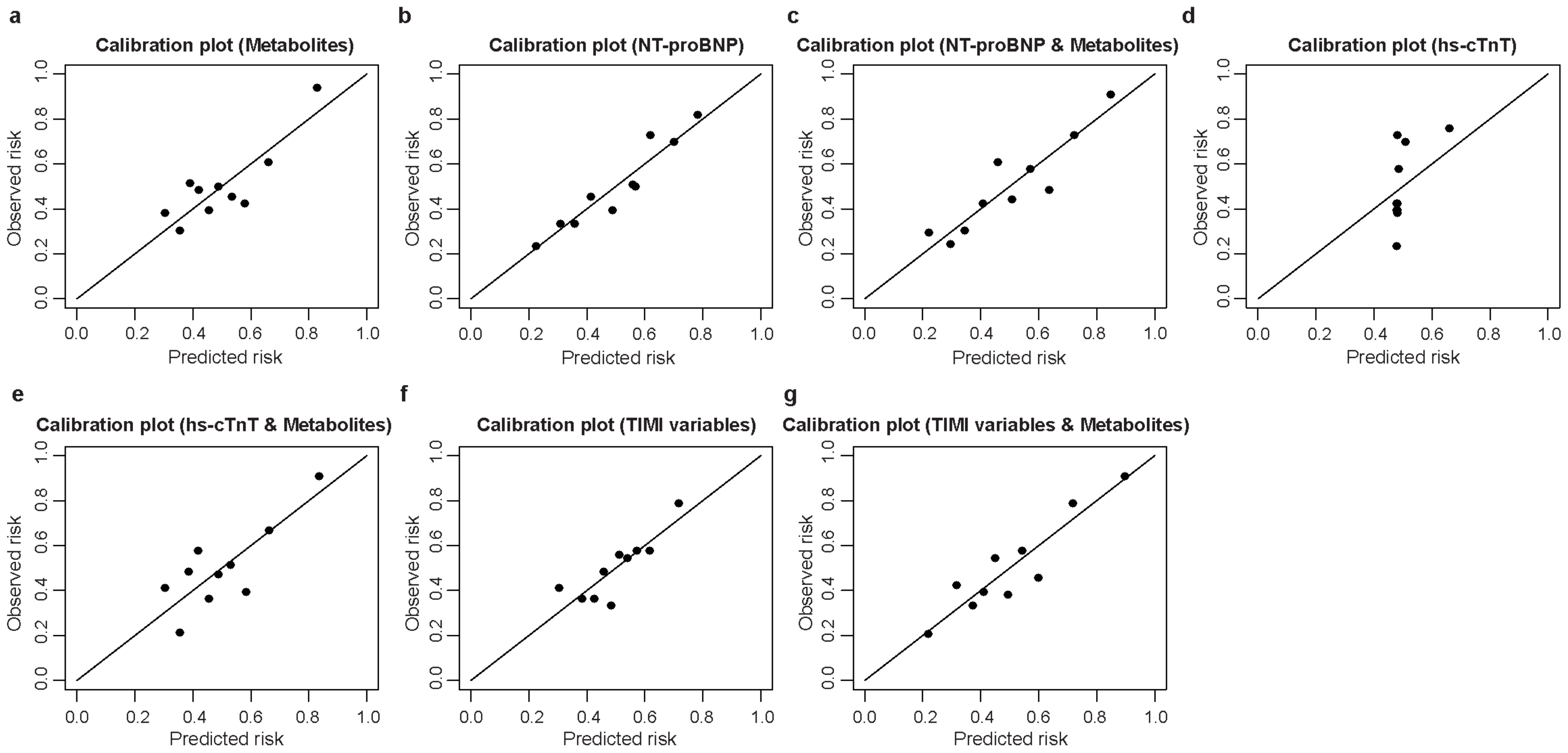

Supplementary Figure 7. The calibration curves of prediction models for the composite of cardiovascular events.

(a) The key metabolites combination; (b) NT-proBNP; (c) NT-proBNP & Metabolites; (d) hs-cTnT; (e) hs-cTnT & Metabolites; (f) TIMI variables; (g) TIMI variables & Metabolites.

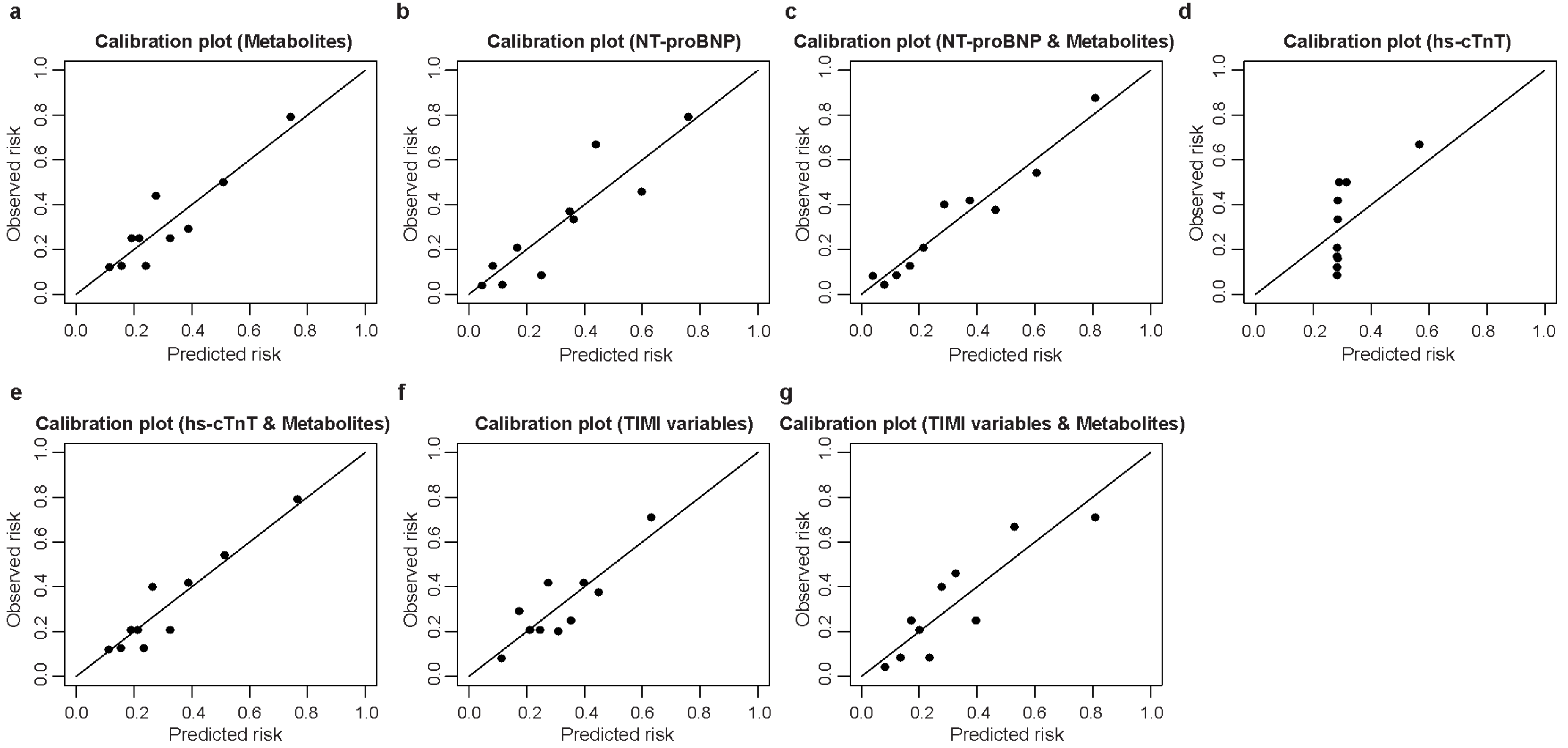

Supplementary Figure 8. The calibration curves of prediction models for cardiovascular death.

(a) The key metabolites combination; (b) NT-proBNP; (c) NT-proBNP & Metabolites; (d) hs-cTnT; (e) hs-cTnT & Metabolites; (f) TIMI variables; (g) TIMI variables & Metabolites.

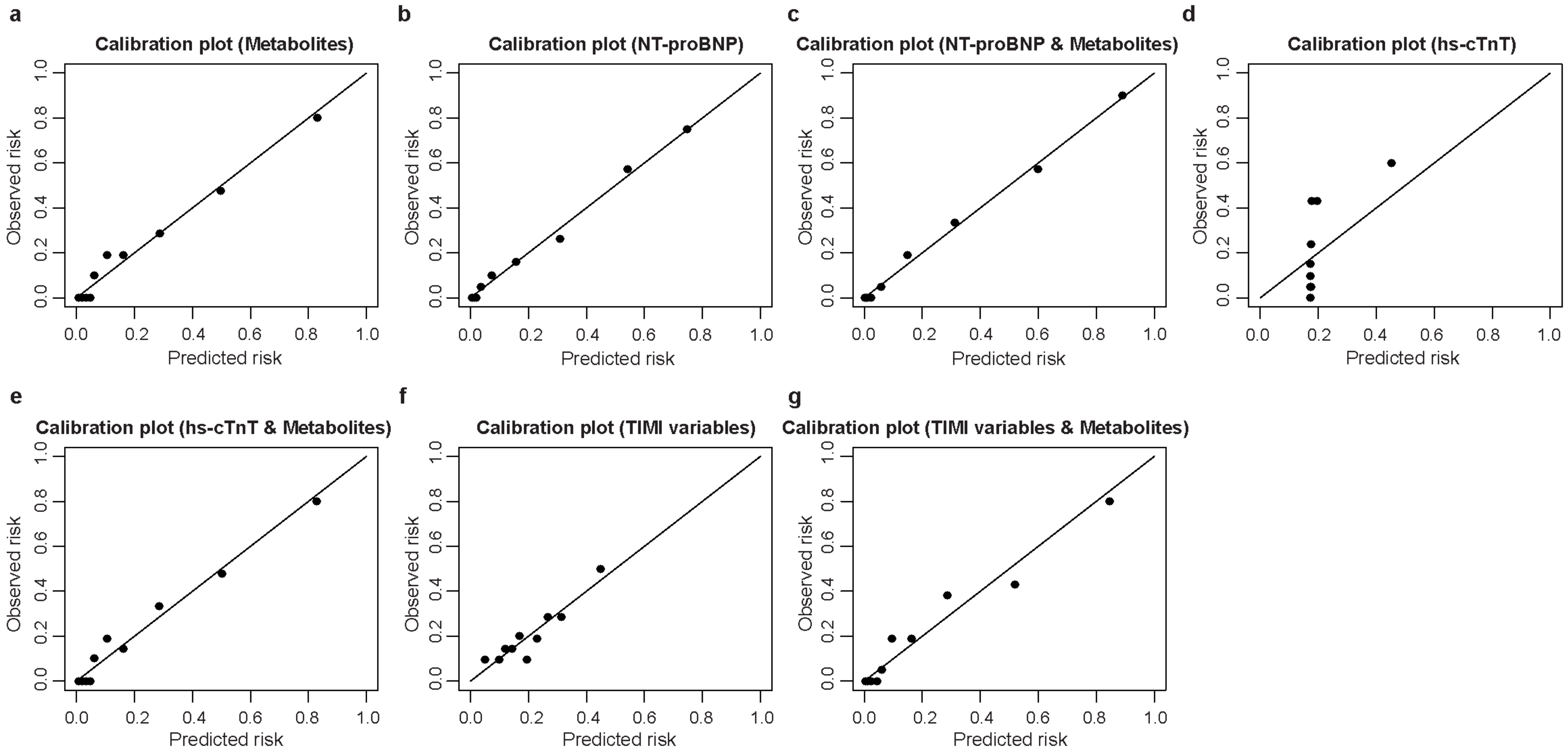

Supplementary Figure 9. The calibration curves of prediction models for heart failure.

(a) The key metabolites combination; (b) NT-proBNP; (c) NT-proBNP & Metabolites; (d) hs-cTnT; (e) hs-cTnT & Metabolites; (f) TIMI variables; (g) TIMI variables & Metabolites.

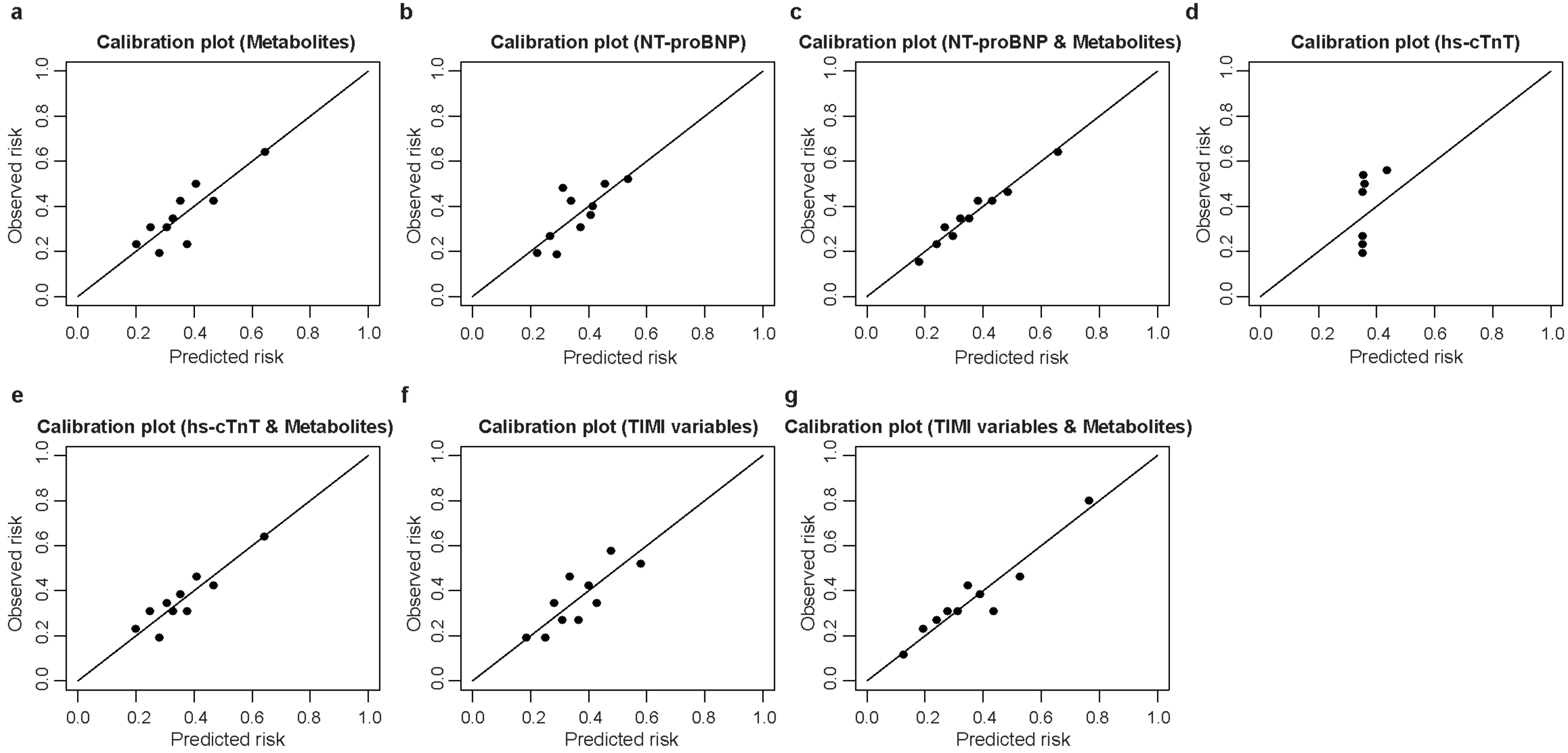

Supplementary Figure 10. The calibration curves of prediction models for myocardial infarction/stroke.

(a) The key metabolites combination; (b) NT-proBNP; (c) NT-proBNP & Metabolites; (d) hs-cTnT; (e) hs-cTnT & Metabolites; (f) TIMI variables; (g) TIMI variables & Metabolites.

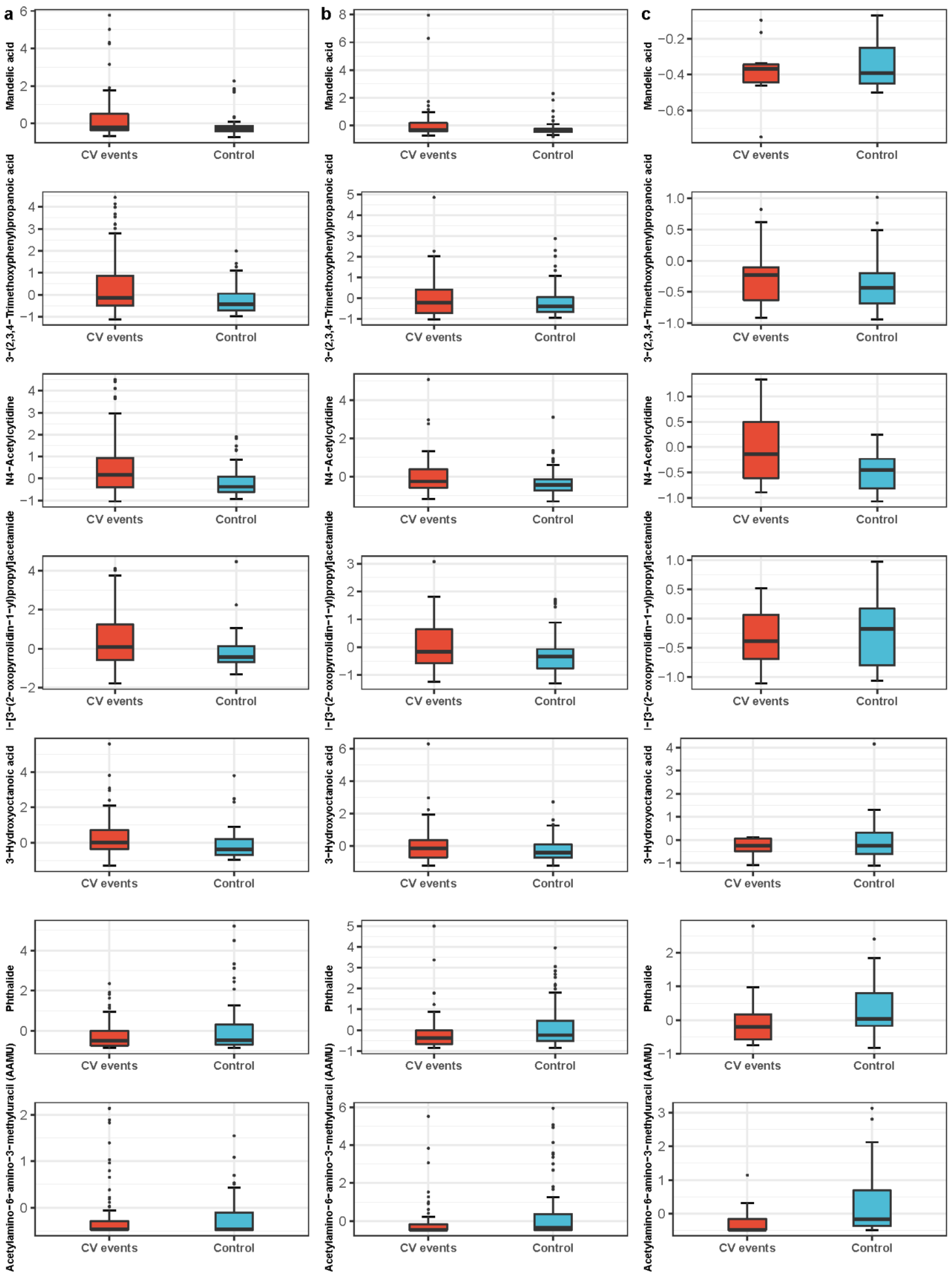

Supplementary Figure 11 (continued on next page). The distribution of the key metabolites combination for the composite of cardiovascular events across different disease subtypes.

(a) Myocardial infarction; (b) Unstable angina; (c) Stable angina.

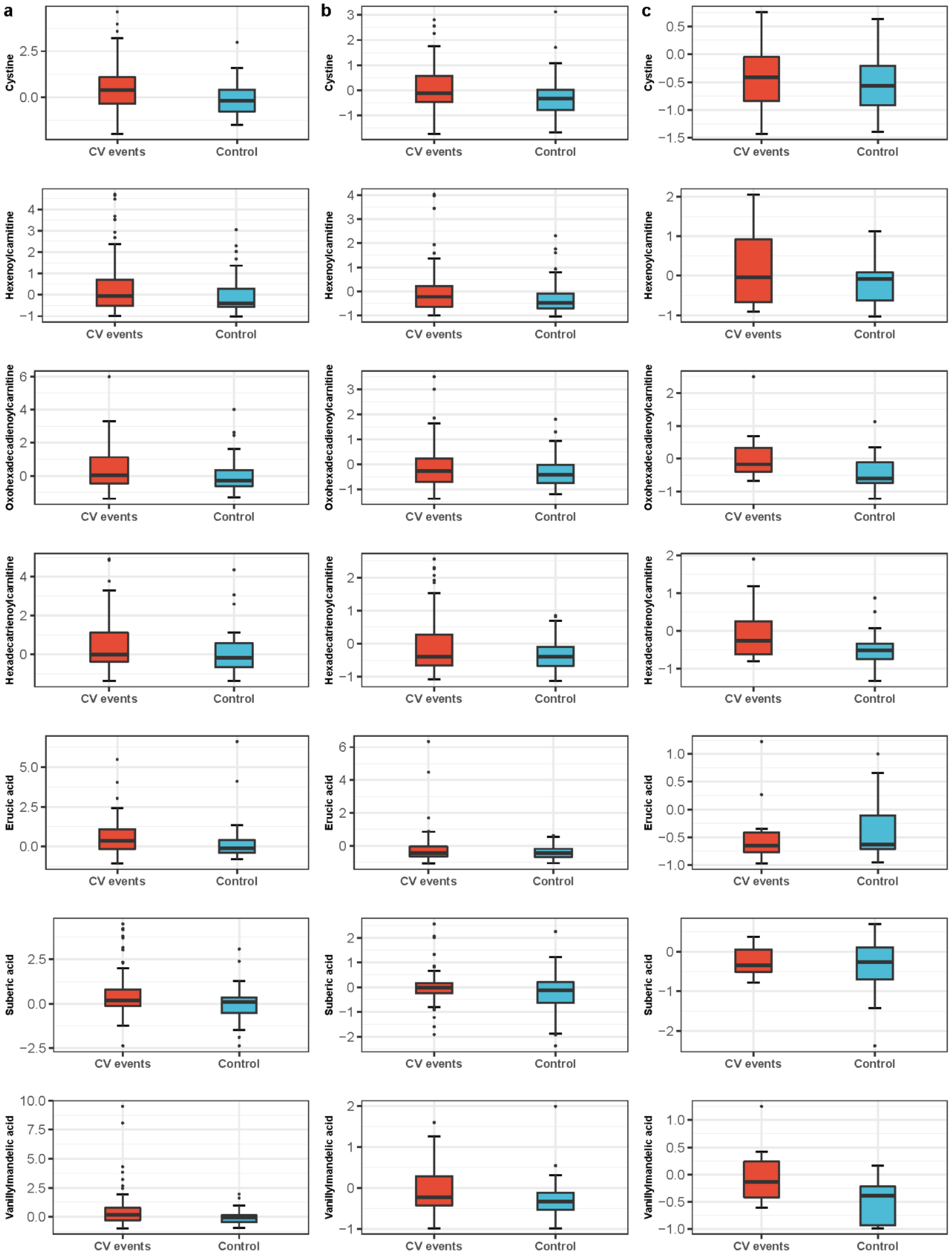

Supplementary Figure 11 (continued). The distribution of the key metabolites combination for the composite of cardiovascular events across different disease subtypes.

(a) Myocardial infarction; (b) Unstable angina; (c) Stable angina.

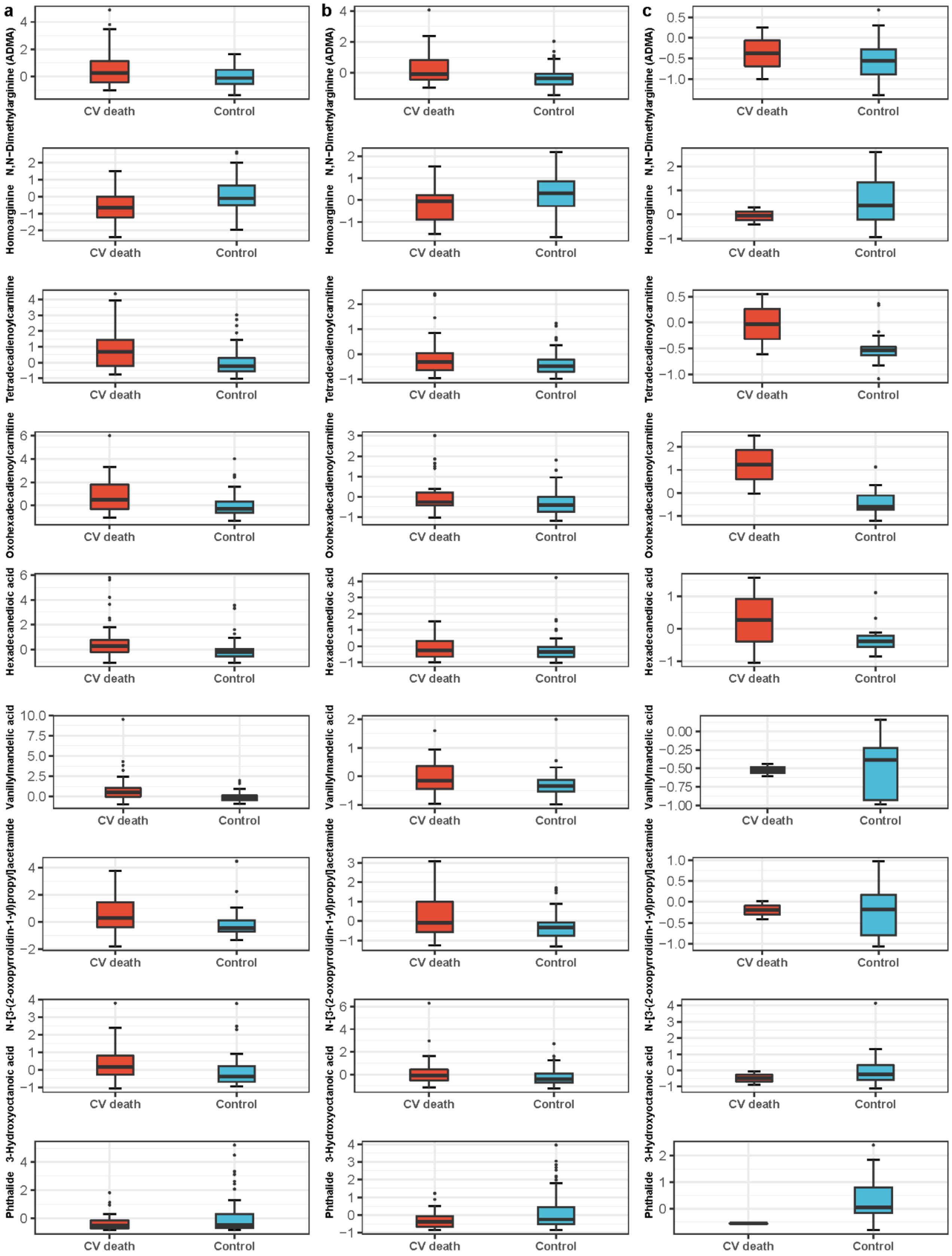

Supplementary Figure 12. The distribution of the key metabolites combination for cardiovascular death across different disease subtypes.

(a) Myocardial infarction; (b) Unstable angina; (c) Stable angina.

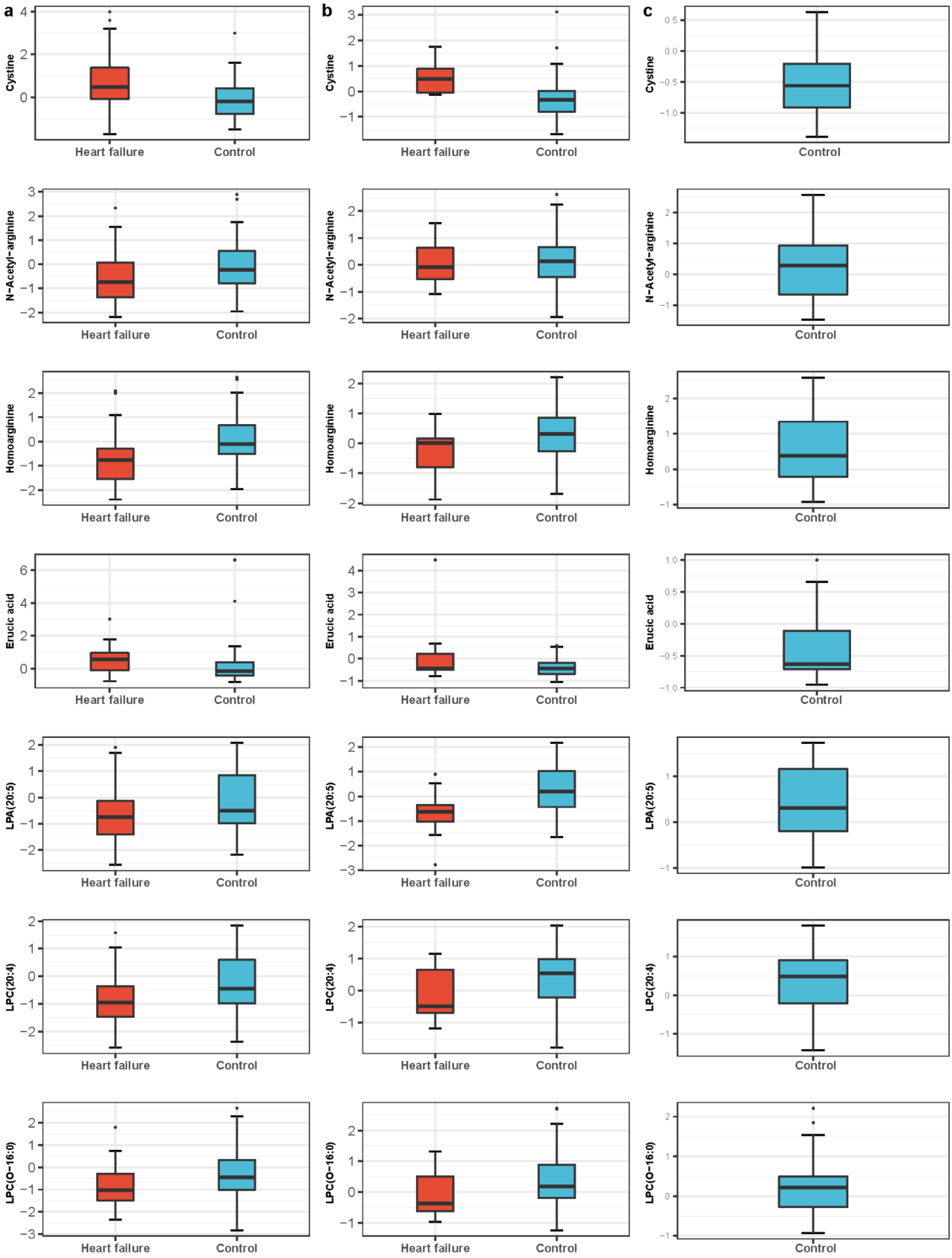

Supplementary Figure 13 (continued on next page). The distribution of the key metabolites combination for heart failure across different disease subtypes.

(a) Myocardial infarction; (b) Unstable angina; (c) Stable angina.

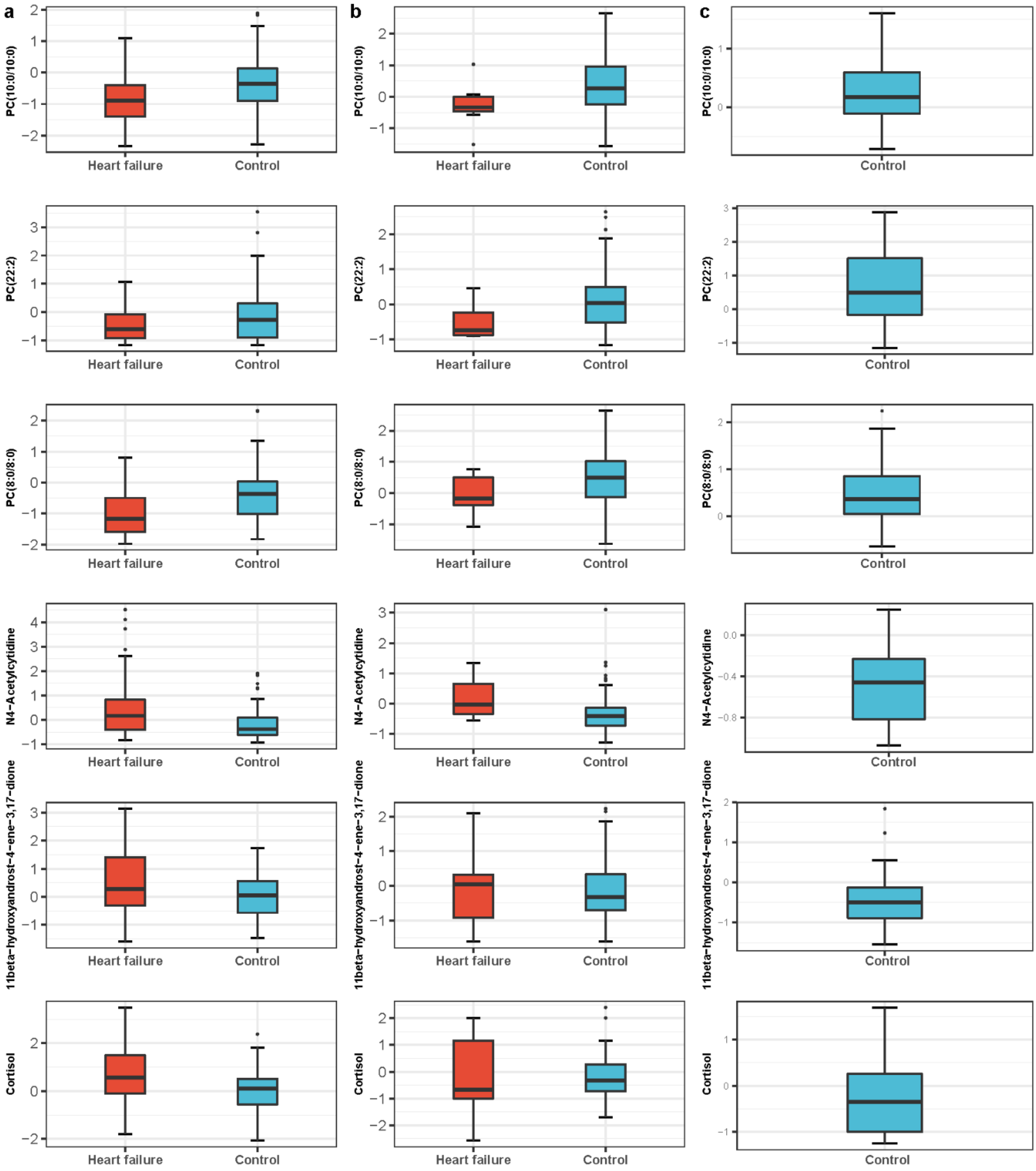

Supplementary Figure 13 (continued). The distribution of the key metabolites combination for heart failure across different disease subtypes.

(a) Myocardial infarction; (b) Unstable angina; (c) Stable angina.

Note: Due to the limitation of sample size, after subdividing the study population, the stable angina subgroup did not report any incident heart failure throughout the follow-up period. Consequently, the figure only depicts the distribution of metabolites within the control group.

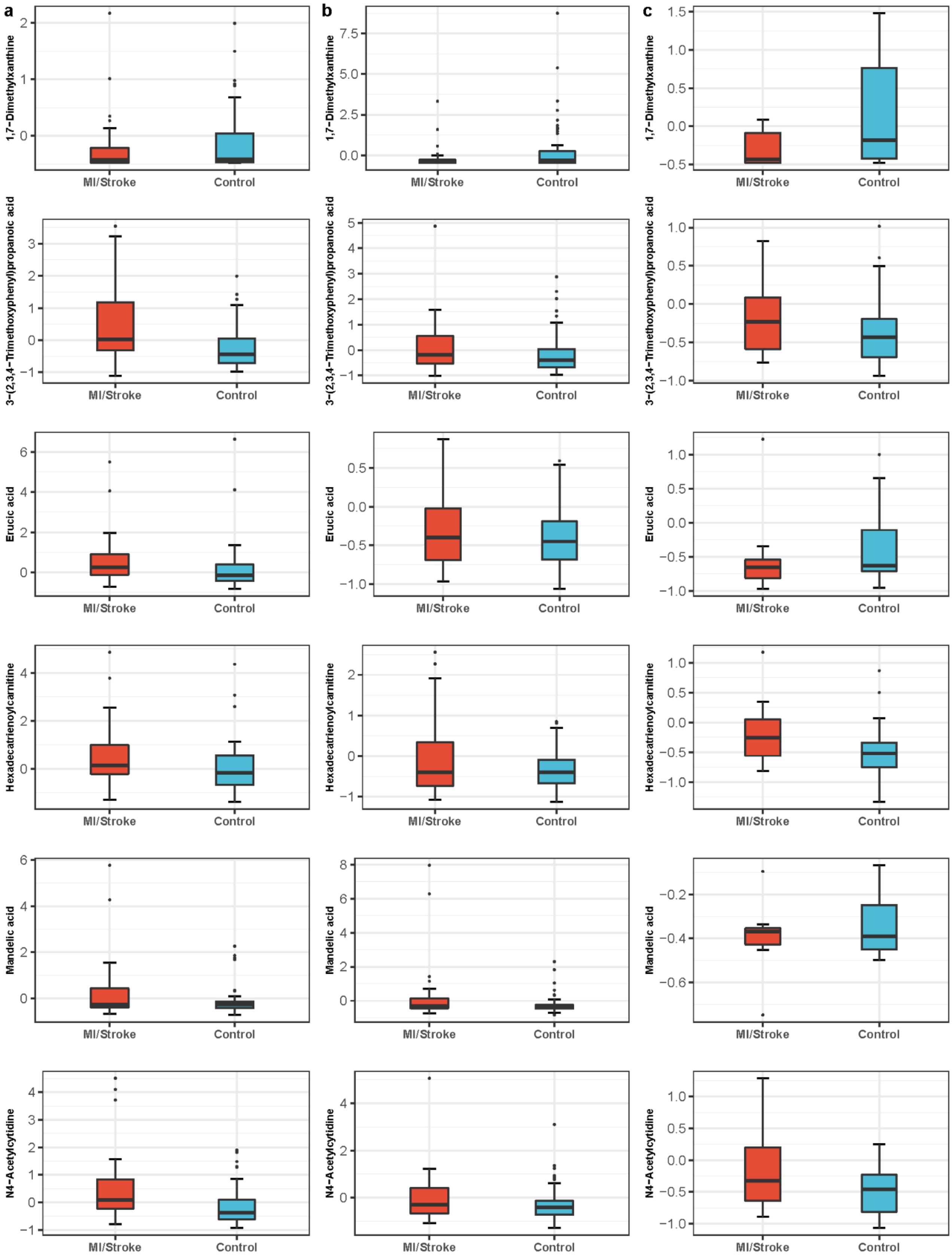

Supplementary Figure 14 (continued on next page). The distribution of the key metabolites combination for myocardial infarction/stroke across different disease subtypes.

(a) Myocardial infarction; (b) Unstable angina; (c) Stable angina.

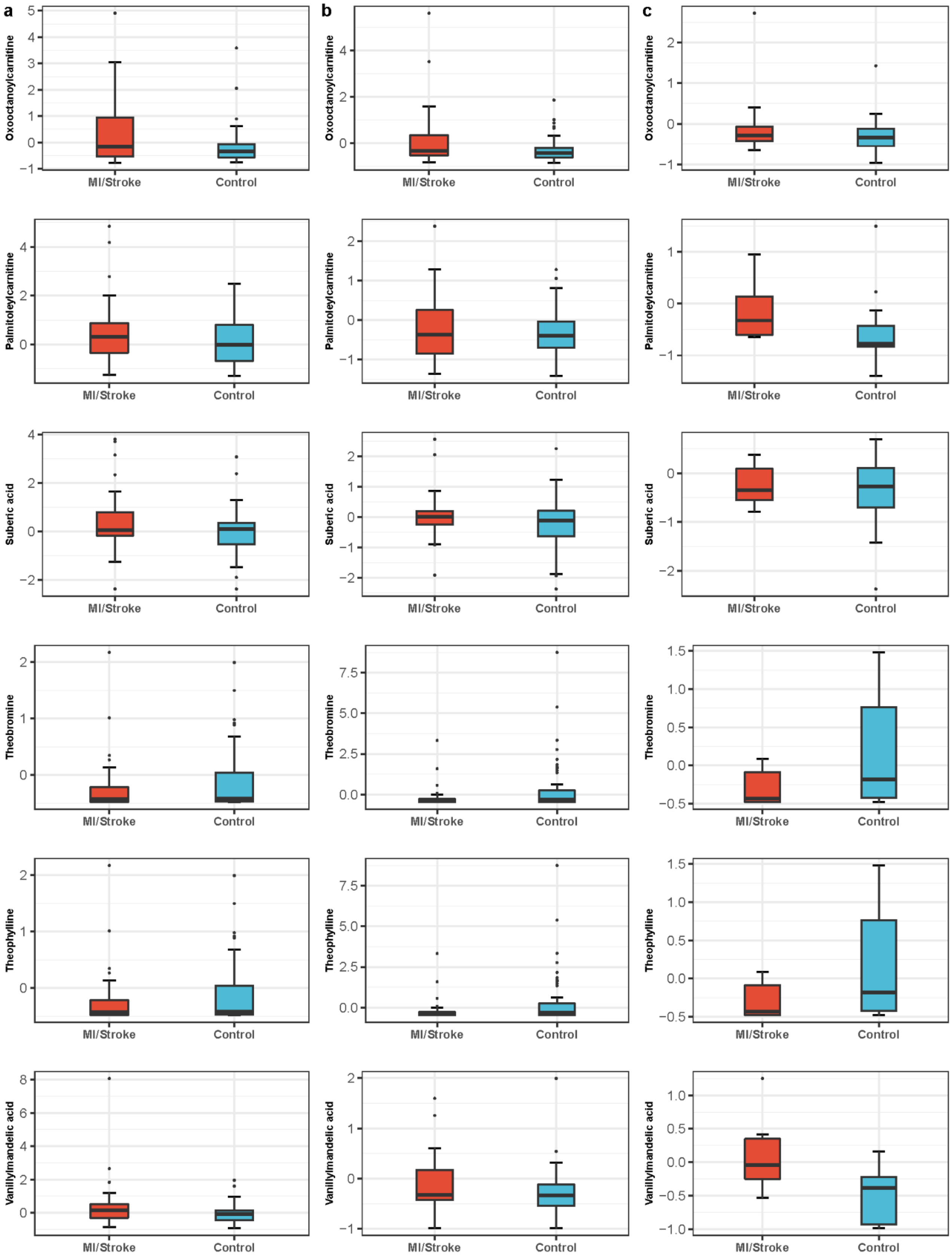

Supplementary Figure 14 (continued). The distribution of the key metabolites combination for myocardial infarction/stroke across different disease subtypes.

(a) Myocardial infarction; (b) Unstable angina; (c) Stable angina.

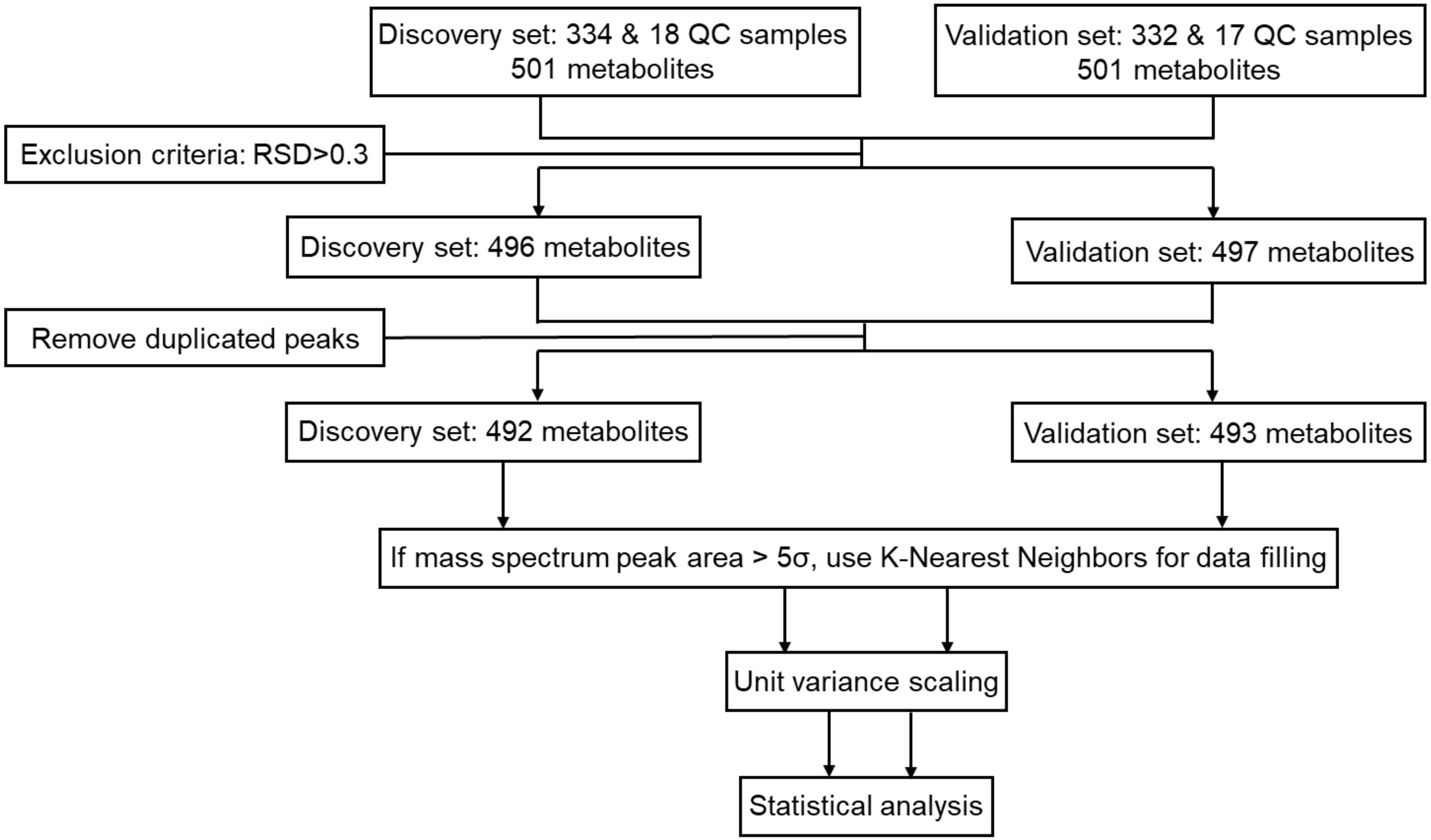

Supplementary Figure 15. The flow chart of metabolomics data processing.

QC = quality control; RSD = relative standard deviation.

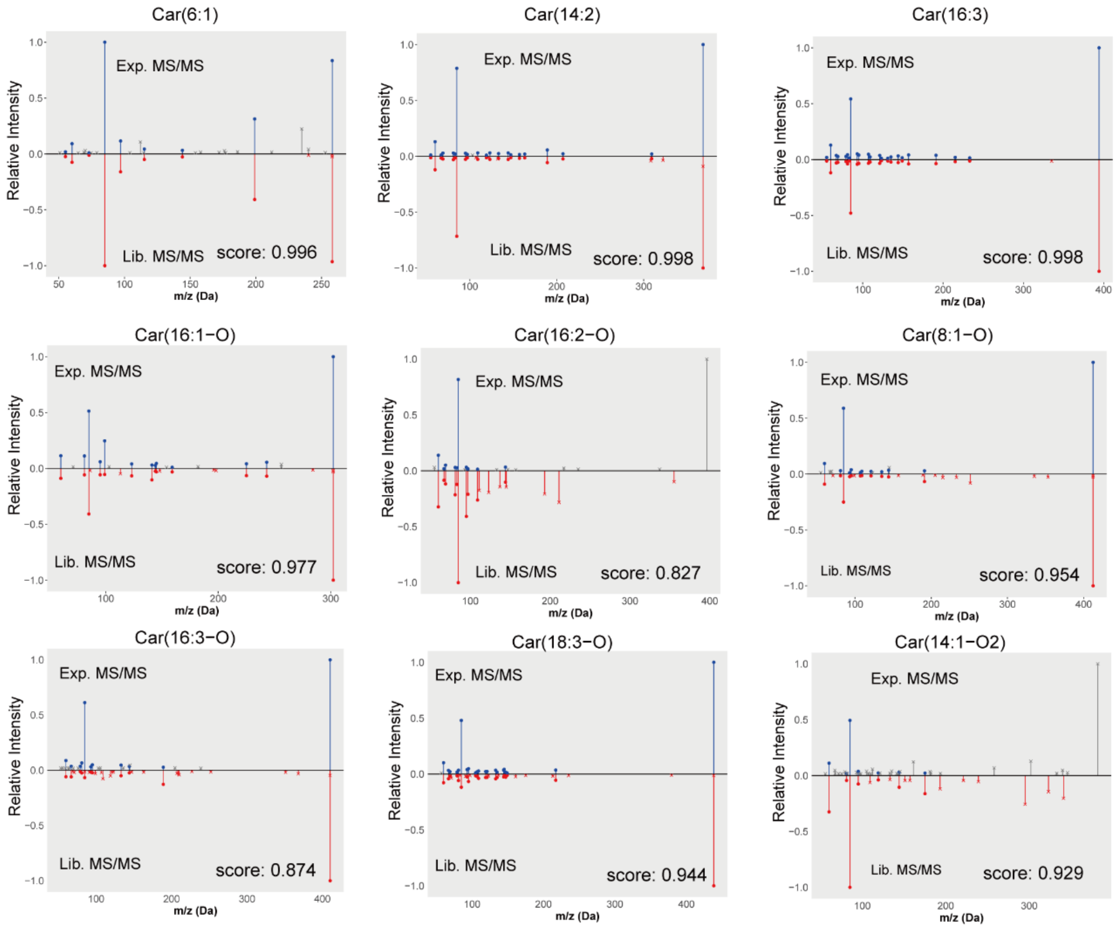

Supplementary Figure 16. Mirror plots of MS/MS match for acylcarnitines.

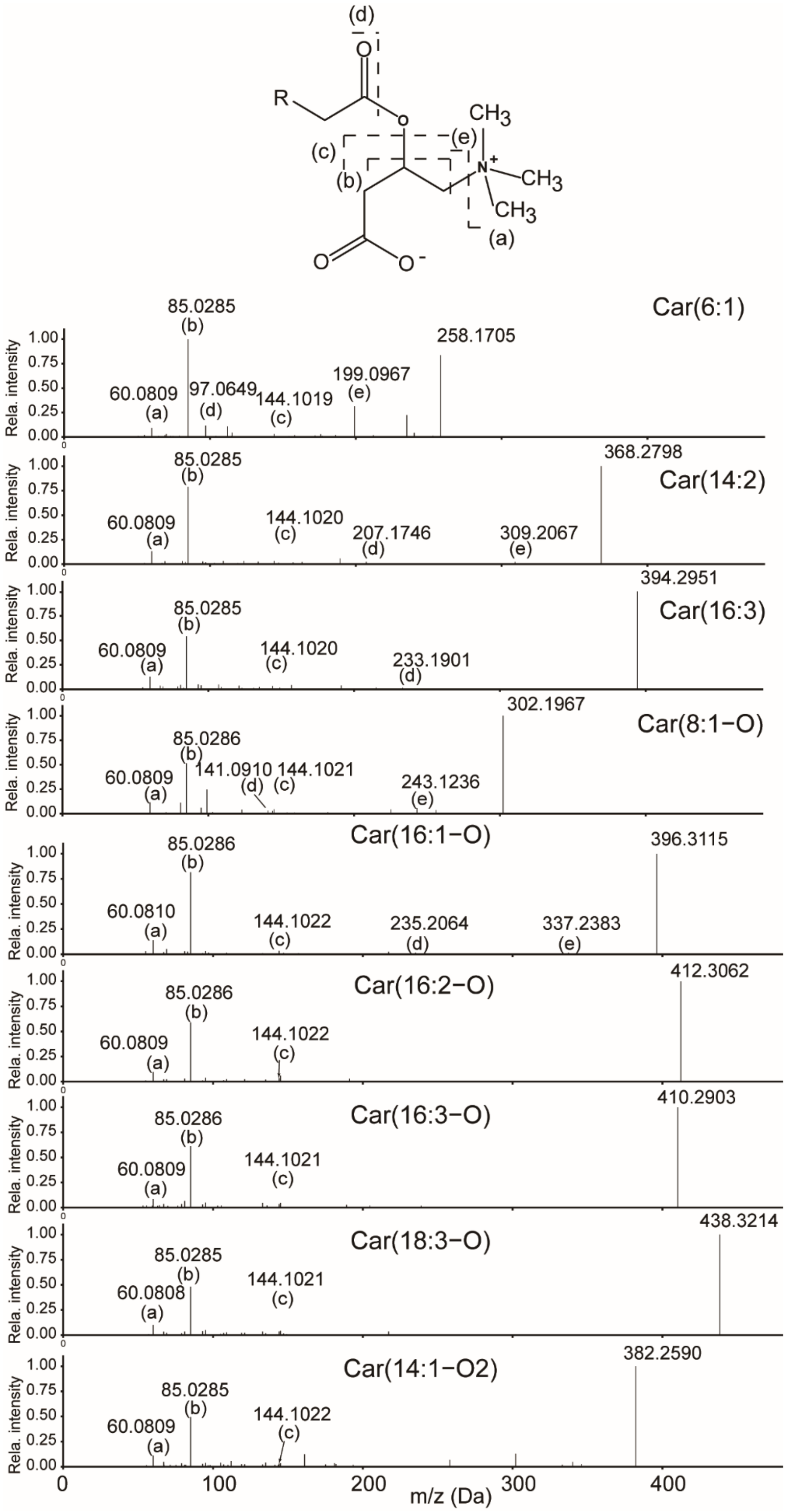

Supplementary Figure 17. Annotation of characteristic fragment ions in mass spectra of acylcarnitines.

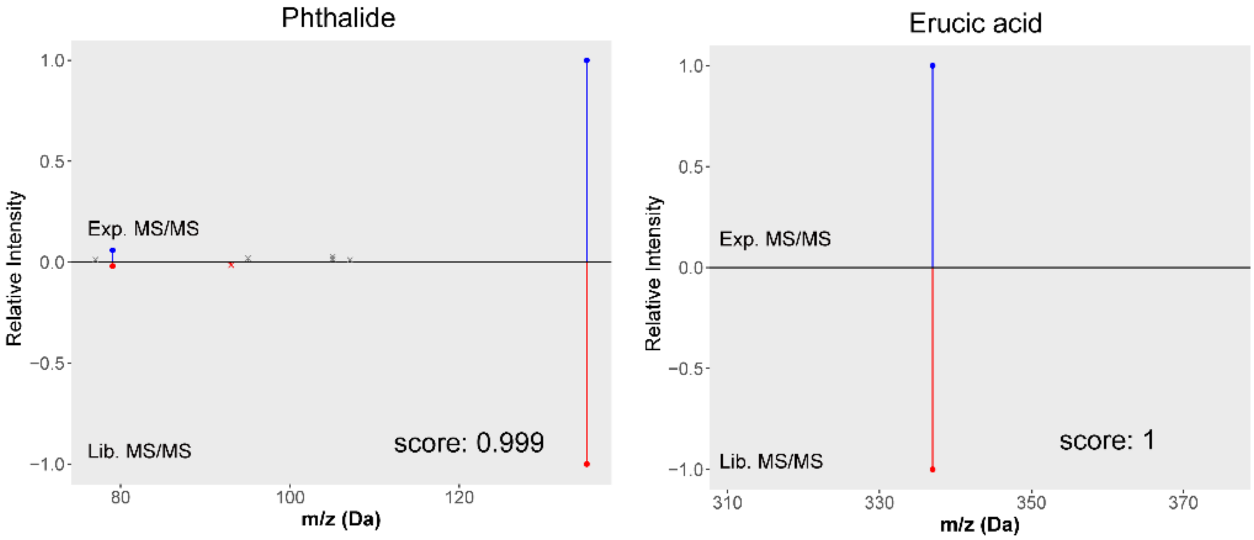

Supplementary Figure 18. Mirror plots for MS/MS spectral match for phthalide and erucic acid.
