# Supplementary material for: Plasma metabolomics reveals the shared and distinct metabolic disturbances associated with cardiovascular events in coronary artery disease
Source: Nat Commun. 2024 Jul 8;15:5729. doi: 10.1038/s41467-024-50125-2 (PMC11231153; doi:10.1038/s41467-024-50125-2)
Supplement: Supplementary file 3 — Description of Additional Supplementary Items [file 41467_2024_50125_MOESM3_ESM.docx]

**Description of Additional Supplementary Files**

**Title**: Supplementary Data 1

**Description**: Clinical characteristics of patients with cardiovascular death.

**Title**: Supplementary Data 2

**Description**: Clinical characteristics of patients with heart failure.

**Title**: Supplementary Data 3

**Description**: Clinical characteristics of patients with myocardial infarction/stroke.

**Title**: Supplementary Data 4

**Description**: KEGG Pathway enrichment analysis for the composite of cardiovascular events.

**Title**: Supplementary Data 5

**Description**: Differential metabolites for the composite of cardiovascular events.

**Title**: Supplementary Data 6

**Description**: Differential metabolites for cardiovascular death.

**Title**: Supplementary Data 7

**Description**: Differential metabolites for heart failure.

**Title**: Supplementary Data 8

**Description**: Differential metabolites for myocardial infarction/stroke.

**Title**: Supplementary Data 9

**Description**: KEGG Pathway enrichment analysis for cardiovascular death.

**Title**: Supplementary Data 10

**Description**: KEGG Pathway enrichment analysis for heart failure.

**Title**: Supplementary Data 11

**Description**: KEGG Pathway enrichment analysis for myocardial infarction/stroke.

**Title**: Supplementary Data 12

**Description**: The associations between differential metabolites and cardiovascular events.

**Title**: Supplementary Data 13

**Description**: The key metabolites combination in prediction models for cardiovascular events.

**Title**: Supplementary Data 14

**Description**: The definitions and diagnostic criteria for stable angina and acute coronary syndromes.

**Title**: Supplementary Data 15

**Description**: The definitions of end points.

**Title**: Supplementary Data 16

**Description**: Detailed information on 492 identified metabolites.
