# Supplementary material for: Plasma metabolomics reveals the shared and distinct metabolic disturbances associated with cardiovascular events in coronary artery disease
Source: Nat Commun. 2024 Jul 8;15:5729. doi: 10.1038/s41467-024-50125-2 (PMC11231153; doi:10.1038/s41467-024-50125-2)
Supplement: Supplementary file 5 — Reporting Summary [file 41467_2024_50125_MOESM5_ESM.pdf]

nature portfolio

Corresponding author(s): ZJZ, TZ, JLW, YGC

Last updated by author(s): May 9, 2024

Reporting Summary

Nature Portfolio wishes to improve the reproducibility of the work that we publish. This form provides structure for consistency and transparency in reporting. For further information on Nature Portfolio policies, see our Editorial Policies and the Editorial Policy Checklist.

Statistics

For all statistical analyses, confirm that the following items are present in the figure legend, table legend, main text, or Methods section.

n/a	Confirmed	
☐	☒	The exact sample size (*n*) for each experimental group/condition, given as a discrete number and unit of measurement
☐	☒	A statement on whether measurements were taken from distinct samples or whether the same sample was measured repeatedly
☐	☒	The statistical test(s) used AND whether they are one- or two-sided<br>*Only common tests should be described solely by name; describe more complex techniques in the Methods section.*
☐	☒	A description of all covariates tested
☐	☒	A description of any assumptions or corrections, such as tests of normality and adjustment for multiple comparisons
☐	☒	A full description of the statistical parameters including central tendency (e.g. means) or other basic estimates (e.g. regression coefficient) AND variation (e.g. standard deviation) or associated estimates of uncertainty (e.g. confidence intervals)
☐	☒	For null hypothesis testing, the test statistic (e.g. *F*, *t*, *r*) with confidence intervals, effect sizes, degrees of freedom and *P* value noted<br>*Give P values as exact values whenever suitable.*
☒	☐	For Bayesian analysis, information on the choice of priors and Markov chain Monte Carlo settings
☐	☒	For hierarchical and complex designs, identification of the appropriate level for tests and full reporting of outcomes
☐	☒	Estimates of effect sizes (e.g. Cohen's *d*, Pearson's *r*), indicating how they were calculated

Our web collection on statistics for biologists contains articles on many of the points above.

Software and code

Policy information about availability of computer code

Data collection	No commercial, open source or custom code were used to collect the data in this study.
Data analysis	All analyses were conducted using the R platform (version 3.6.0). Besides, we conducted pathway enrichment analyses using MetaboAnalyst 6.0 (https://www.metaboanalyst.ca/), which incorporated the Kyoto Encyclopedia of Genes and Genomes (KEGG) pathway database (accessed in December 2023). Metabolomics data were processed using XCMS (version 3.2) and MetDNA (version 1.2.2; http://metdna.zhulab.cn/). We ascertained the classification of these metabolites by cross-referencing each metabolite with the Human Metabolome Database (HMDB, https://hmdb.ca/). To identify clusters within these metabolic networks, we applied the walktrap-algorithm using the igraph R-package (https://igraph.org/) and Gephi (version 0.10.1). Powers of the Wilcoxon-Mann-Whitney test were evaluated by the G*Power software (version 3.1.9.7). Portions of figure 1 were created with BioRender.com.

For manuscripts utilizing custom algorithms or software that are central to the research but not yet described in published literature, software must be made available to editors and reviewers. We strongly encourage code deposition in a community repository (e.g. GitHub). See the Nature Portfolio guidelines for submitting code & software for further information.

Data

Policy information about availability of data

All manuscripts must include a data availability statement. This statement should provide the following information, where applicable:

- Accession codes, unique identifiers, or web links for publicly available datasets
- A description of any restrictions on data availability
- For clinical datasets or third party data, please ensure that the statement adheres to our policy

All the participant data for this study, including the individual participant data and a data dictionary for each variable in the study, will be shared upon request for the corresponding author Dr. Yuguo Chen. Associated code was available on the Code Ocean website (https://codeocean.com/capsule/8925883/tree).

Research involving human participants, their data, or biological material

Policy information about studies with human participants or human data. See also policy information about sex, gender (identity/presentation), and sexual orientation and race, ethnicity and racism.

Reporting on sex and gender	The sex information in this study was determined by biological attribute. Among the 666 CAD patients, a total of 255 (38.3%) females were included in this study, with 121 (47.5%) females developed the composite of cardiovascular events. We also observed 212 (51.6%) males developed the composite of cardiovascular events during follow-up. Considering the issue of sample size, we did not conduct post hoc sex-based analysis in this study.
Reporting on race, ethnicity, or other socially relevant groupings	All the subjects included in this study were Asian yellow race. We adopted the following measures to control confounding variables in our analyses: 1) In the research design stage, we randomly selected the controls from all participants at risk, matched by age, sex, body mass index (BMI), current smoking, hypertension, diabetes, and previous MI, according to a 1:1 propensity score matching at group level; 2) When evaluating the association of differential metabolites with cardiovascular events, we have adjusted TIMI variables (age, current smoking, hypertension, diabetes mellitus, previous stroke, previous HF, previous PAD, previous PCI/CABG, and eGFR), hs-cTnT and NT-proBNP. In the revision, We further adjusted pre-hospital medical treatments (β-receptor blockers, ACEI/ARB, statins, and aspirin), the severity of coronary stenosis and different clinical phenotypes (systolic blood pressure, heart rate, and admission diagnosis).
Population characteristics	The 666 CAD patients had a median age of 68.2 years (interquartile range 61.7~74.8years), with 255 (38.3%) female, the median body-mass index was 24.8 kg/m2 (interquartile range 22.9~27.2 kg/m2), the median systolic blood pressure 134 mmHg (interquartile range 122~150 mmHg), and the median low density lipoprotein-cholesterol level 2.2 interquartile range 1.8~2.8 mmol/L); 19.7% were current smokers and 44.4% had diabetes. Patients with the composite of cardiovascular events demonstrated higher levels of heart rates, white blood cell counts, creatinine, cystain C, hs-cTnT, and NT-proBNP, but lower levels of eGFR, and hemoglobin, compared to those without any cardiovascular events.
Recruitment	Patients from two participating hospitals of the BIPass cohort (Site 1: Qilu hospital of Shandong University, Jinan, China; Site 2: Zibo Central hospital, Zibo, China) were included in this study. The inclusion and exclusion criteria of patients have been described in Supplementary Table 5.
Ethics oversight	This study was approved by the research ethics committee of Qilu Hospital of Shandong University, and accepted by Zibo Central hospital.

Note that full information on the approval of the study protocol must also be provided in the manuscript.

Field-specific reporting

Please select the one below that is the best fit for your research. If you are not sure, read the appropriate sections before making your selection.

☒ Life sciences ☐ Behavioural & social sciences ☐ Ecological, evolutionary & environmental sciences

For a reference copy of the document with all sections, see nature.com/documents/nr-reporting-summary-flat.pdf

Life sciences study design

All studies must disclose on these points even when the disclosure is negative.

Sample size	Powers of the Wilcoxon-Mann-Whitney test were evaluated by the G*Power software (version 3.1.9.7) and a t-test with two tails specific for the Wilcoxon-Mann-Whitney test (two groups) was used. With the α level set at 0.05 and the effect size (d) of 0.5, the statistical power for the composite of cardiovascular events (control 167 vs case 167), cardiovascular death (control 167 vs case 82), heart failure (control 167 vs case 48), and myocardial infarction/stroke (control 167 vs case 72) were found to be 0.994, 0.950, 0.844, and 0.932, respectively. Based on these results, we believe that our current sample size is sufficient to support the discovery of differential metabolic biomarkers.
Data exclusions	1. Previous surgery, trauma or clinically evident coagulopathic bleeding (i.e. gastrointestinal, genitourinary) within the prior 2 weeks;<br>2. Bipass population with missing follow-up records in Qilu Hospital emergency follow-up cohort.
Replication	Sensitivity analyses were performed in the revised manuscript, excluding patients admitted with stable angina, and all analyses were repeated only in acute coronary syndrome patients. These results were consistent with the conclusions in our manuscript. In addition, we also showed

	the distribution of differential metabolites in patients admitted with myocardial infarction, unstable angina, and stable angina. The boxplots showed that the distribution of differential metabolites in different populations was still stable. The above results confirmed that these results were replicated.
Randomization	In this study, we conducted a nested case-control design, including 333 patients with incident cardiovascular events as case group and 333 patients without any events as control group. The controls were randomly selected from all participants at risk, matched by age, sex, body mass index (BMI), current smoking, hypertension, diabetes, and previous MI, according to a 1:1 propensity score matching at group level.
Blinding	We declare that the investigators conducting untargeted metabolomics analyses were blinded to group allocation.

Reporting for specific materials, systems and methods

We require information from authors about some types of materials, experimental systems and methods used in many studies. Here, indicate whether each material, system or method listed is relevant to your study. If you are not sure if a list item applies to your research, read the appropriate section before selecting a response.

Materials & experimental systems

n/a	Involved in the study
☒	☐ Antibodies
☒	☐ Eukaryotic cell lines
☒	☐ Palaeontology and archaeology
☒	☐ Animals and other organisms
☐	☒ Clinical data
☒	☐ Dual use research of concern
☒	☐ Plants

Methods

n/a	Involved in the study
☒	☐ ChIP-seq
☒	☐ Flow cytometry
☒	☐ MRI-based neuroimaging

Clinical data

Policy information about clinical studies

All manuscripts should comply with the ICMJE guidelines for publication of clinical research and a completed CONSORT checklist must be included with all submissions.

Clinical trial registration	NCT05550805
Study protocol	https://classic.clinicaltrials.gov/ct2/show/NCT05550805
Data collection	Patients from two participating hospitals of the BIPass cohort (Site 1: Qilu hospital of Shandong University, Jinan, China; Site 2: Zibo Central hospital, Zibo, China) were included in this study. Patients from site 1 were first randomly assigned to the discovery set (n = 334, 167 with cardiovascular events and 167 without any cardiovascular event), and the remained patients were assigned to the validation set (n = 240, 123 with cardiovascular events and 117 without any cardiovascular event).
Outcomes	In this study, we identified the composite of cardiovascular events as the primary endpoint and cardiovascular death, HF and MI/stroke as secondary endpoints. Detailed definitions of end points were provided Supplementary Data 15.

Plants

Seed stocks	*Report on the source of all seed stocks or other plant material used. If applicable, state the seed stock centre and catalogue number. If plant specimens were collected from the field, describe the collection location, date and sampling procedures.*
Novel plant genotypes	*Describe the methods by which all novel plant genotypes were produced. This includes those generated by transgenic approaches, gene editing, chemical/radiation-based mutagenesis and hybridization. For transgenic lines, describe the transformation method, the number of independent lines analyzed and the generation upon which experiments were performed. For gene-edited lines, describe the editor used, the endogenous sequence targeted for editing, the targeting guide RNA sequence (if applicable) and how the editor was applied.*
Authentication	*Describe any authentication procedures for each seed stock used or novel genotype generated. Describe any experiments used to assess the effect of a mutation and, where applicable, how potential secondary effects (e.g. second site T-DNA insertions, mosiacism, off-target gene editing) were examined.*
